# Local level epidemiological analysis of TB in people from a high incidence country of birth

**DOI:** 10.1186/1471-2458-13-62

**Published:** 2013-01-22

**Authors:** Peter D Massey, David N Durrheim, Nicola Stephens, Amanda Christensen

**Affiliations:** 1 Hunter New England Population Health, Locked bag 9783, NEMSC, Tamworth, NSW 2340, Australia; 2 Hunter Medical Research Institute, Lot 1 Kookaburra Circuit, New Lambton Heights, NSW, 2305, Australia; 3 Health Protection Branch, Department of Health, 14/50 Lonsdale Street, Melbourne, Victoria, 3000, Australia; 4 Health Protection NSW, Locked Mail Bag 961, North Sydney, NSW, 2059, Australia

**Keywords:** Tuberculosis, Epidemiology, Low-incidence setting, Country-of-birth

## Abstract

**Background:**

The setting for this analysis is the low tuberculosis (TB) incidence state of New South Wales (NSW), Australia. Local level analysis of TB epidemiology in people from high incidence countries-of-birth (HIC) in a low incidence setting has not been conducted in Australia and has not been widely reported. Local level analysis could inform measures such as active case finding and targeted earlier diagnosis. The aim of this study was to use a novel approach to identify local areas in an Australian state that have higher TB rates given the local areas’ country of birth profiles.

**Methods:**

TB notification data for the three year period 2006–2008 were analysed by grouping the population into those from a high-incidence country-of-birth and the remainder.

**Results:**

During the study period there were 1401 notified TB cases in the state of NSW. Of these TB cases 76.5% were born in a high-incidence country. The annualised TB notification rate for the high-incidence country-of-birth group was 61.2/100,000 population and for the remainder of the population was 1.8/100,000. Of the 152 Local Government Areas (LGA) in NSW, nine had higher and four had lower TB notification rates in their high-incidence country-of-birth populations when compared with the high-incidence country-of-birth population for the rest of NSW. The nine areas had a higher proportion of the population with a country of birth where TB notification rates are >100/100,000. Those notified with TB in the nine areas also had a shorter length of stay in Australia than the rest of the state. The areas with higher TB notification rates were all in the capital city, Sydney. Among LGAs with higher TB notification rates, four had higher rates in both people with a high-incidence country of birth and people not born in a high-incidence country. The age distribution of the HIC population was similar across all areas, and the highest differential in TB rates across areas was in the 5–19 years age group.

**Conclusions:**

Analysing local area TB rates and possible explanatory variables can provide useful insights into the epidemiology of TB. TB notification rates that take country of birth in local areas into account could enable health services to strategically target TB control measures.

## Background

In many low incidence countries such as Australia, Canada, New Zealand and the United Kingdom, higher rates of tuberculosis (TB) are reported in recent immigrants [1-4]. For example the increasing TB rate in the United Kingdom has been considered a result of increased notifications in migrants from countries with a high TB incidence [5]. In these settings TB incidence continues to decline in locally born people, resulting in a higher proportion of foreign-born cases [6,7].

The TB incidence in the country of birth is the most important population level predictor of TB rates among migrants in Australia and Canada [8,9]. Among migrants of high-incidence country of birth (HIC), the numbers of TB notifications is highest within the first few years of arrival to a low incidence country, remains higher for seven to ten years, and then decreases substantially in subsequent years [1,7]. In contrast, very few people migrating from lower-incidence countries to Australia and New Zealand are notified with TB within 1 year of arrival [7,10].

Reports from an urban area in the United Kingdom suggest that services that do not take account of ethnic mix may result in delayed diagnosis and poor clinical outcomes [11,12]. Local level epidemiological analysis of TB occurrence in people from a HIC in a low incidence setting may enable health services to target local TB control measures [12,13]. This type of local level analysis has not been conducted in Australia. Local level analysis could inform measures such as active case finding and low threshold diagnostic services to facilitate earlier diagnosis in the targeted areas [13].

Epidemiological reports of TB in low incidence countries such as Australia [1,14], Canada [2] and New Zealand [3] do not routinely report TB rates for local areas or take into account the countries of birth in the local area. Age standardisation is routinely performed on TB rates but this may only partly correct for the risk in the migrant population.

The aim of this study was to use a novel approach to the epidemiology of TB, to identify local areas in an Australian state that have higher rates of TB given the local areas’ country of birth profiles. This analysis will inform further focused epidemiological studies, including if necessary molecular studies.

## Method

The study population is the people of the state of New South Wales (NSW), Australia, for the period 2006–2008. Sydney is the capital city of NSW with more than half of the state’s population.

Population data was sourced from the Australian Bureau of Statistics (ABS) 2006 Census, by Local Government Area (LGA). The LGA is a geographical area under the responsibility of an incorporated local government council. The number of LGAs and their boundaries can change over time. Their creation and delimitation is the responsibility of the respective State/Territory Governments. The LGAs applicable to the 2006 Census and this study are those which existed at 7 August, 2005 [15].

Country of birth data is only available at LGA level for Census years and there are no published estimates for intervening years. Thus the most recent Census (2006) population data was used and multiplied by three to provide the denominator population groups for the three-year study period.

Data on TB is collected in NSW under the requirements of the *Public Health Act (2010)*, with all cases of tuberculosis meeting the case definitions of the National Notifiable Diseases Surveillance System [16]. Case and population data were sourced through the Health Outcomes Information Statistical Toolkit (HOIST). HOIST is a SAS-based 'data warehouse' operated by the Centre for Epidemiology and Research of the NSW Department of Health.

Country of birth data is collected on each notified TB case. For the purposes of this analysis TB cases were defined into two groups: people born in a high TB incidence country and people not born in a high TB incidence country. The population for the state and each LGA were also grouped using the same definition.

We used the New South Wales Health definition of high-incidence, being a country with TB incidence (all forms) being greater than or equal to 60 per 100,000 population per year [17]. It is acknowledged that this definition is different to that used by other jurisdictions. It is the current operational definition used by the health department where the study occured.

Using the three-year study period, 2006–2008, annualised TB rates for the HIC population and the population not born in a HIC, were calculated by LGA and for the state as a whole. A three-year period was used to take account of variations in the annual TB incidence data and the small number of cases in each year.

Annualised crude notifications rates were calculated for each population group at the state level. The rates were compared using a single tailed Fishers Exact Test with exact p-values.

A relative risk of having notified TB was calculated for the HIC population and the remaining population, for the LGAs. The risk was calculated by comparing the LGA TB rate for HIC population and the remaining population, to the TB rates in the corresponding population groups for the state (excluding the LGA population). Using Microsoft Excel, relative risks with 95% confidence intervals were calculated for the HIC population and the remaining population in each LGA. LGAs were classified as significantly higher than NSW if their relative risk was higher than 1.0 and with 95% confidence intervals excluding 1.0. LGAs were classified as significantly lower than NSW if their relative risk was lower than 1.0 and with 95% confidence intervals excluding 1.0.

The relationship between age-distribution and TB incidence in LGAs was explored, using the Pearson’s correlation co-efficient and 95% confidence intervals. Direct age-standardisation was conducted for the combined LGAs identified as having higher rates of TB in the HIC population group. The TB rates in the HIC population were compared to the rest of NSW by age groups 0–4 years, 5–19 years, 20–64 years and 65 years and above. Rate ratios were calculated with 95% confidence intervals.

For the LGAs identified as having higher TB rates given the mix of the local population, further differences in the populations were explored as explanatory variables. The proportions of the population with country of birth TB rates of more than 100/100,000 population and more than 300/100,000 population were compared to the rest of NSW by Chi-square testing with a p-value of less than 0.05 set as significant. In addition the mean length of stay in Australia were compared for the two population groups using Student’s t-test with a p-value of less than 0.05 set as significant.

The Index of Relative Socioeconomic Disadvantage (IRSD) for LGAs of the state was sourced from the ABS Socio-Economic Indexes for Areas through HOIST. The IRSD contains indicators of disadvantage such as low income, high unemployment and low levels of education [18]. A high IRSD score implies less disadvantage for a particular LGA, allowing comparisons to be made [18]. The relationship between the IRSD index and TB incidence in LGAs was explored using the Pearson’s correlation co-efficient. The IRSD does not describe the relative disadvantage of a person, only of the general level of relative disadvantage of people and households in the area.

Analysis was performed using Microsoft Excel® 2010 and SPSS® (Grad Pack 15.0 for Windows).

The study was deemed a quality improvement project by the Hunter New England Health Human Ethics Review Committee.

## Results

During the study period 2006–2008 there were 1401 notified cases of TB in NSW of which 76.5% (n=1071) were born in a high-incidence country. The annualised crude rate of all TB in NSW during the study period was 6.9 /100,000 population. For NSW 8.6% of the population was born in a high-incidence country. The annualised TB rate in people born in a HIC for all of NSW was 62.0/100,000 population, which was significantly higher than the 1.8 / 100,000 population annualised TB rate for the remainder of the population (Table 1).

**Table 1:** Annualised crude rates of TB for NSW, by high incidence country of birth (HIC), 2006-2008

	**Notified TB cases 2006-2008**	**Average Population, 2006-2008**	**Annualized crude notification rate of TB/ 100,000 pop.**	**p-value**
NSW residents born in a HIC	1071	575,537	62.0	<0.0001
All other NSW residents	330	6,231,785	1.8	
Total average	1401	6,807,322	6.9	

There were 152 LGAs in NSW during the study period. Thirty-eight were in the capital city area and 114 were regional or rural.

Nine metropolitan LGAs in the capital city had significantly higher rates of TB (range 77.0 – 116.6 per 100,000 population) among people born in a HIC, compared to people born in a HIC for the rest of NSW. (Table 2) Four of these LGAs also had higher TB rates in the non-HIC population (Table 3).

**Table 2:** Notified TB cases 2006–2008 in high incidence country of birth (HIC) population by Local Government Areas with relative risk >1.0 compared to rest of NSW HIC population

**LGA**	**Total Notified TB cases, 2006-2008**	**Average HIC population, 2006-2008**	**Annualised TB rate HIC**	**RR to rest of NSW**	**95% Confidence Interval (CI)**	
					**Upper**	**Lower**
Metro1	60	21653	92.4	1.57	1.2	2.0
Metro2	21	6140	114.0	1.85	1.2	2.9
Metro3	105	45446	77.0	1.27	1.0*	1.6
Metro4	39	14973	86.8	1.41	1.0*	2.0
Metro5	29	8291	116.6	1.9	1.3	2.8
Metro6	96	32335	99.0	1.65	1.3	2.0
Metro7	38	13914	91.0	1.48	1.1	2.1
Metro8	32	11102	96.1	1.57	1.1	2.2
Metro9	72	24555	97.7	1.62	1.3	2.1
NSW	1071	575537	62.0	-		

* Lower 95%CI 1.03 & 1.04.

**Table 3:** Notified TB cases 2006–2008 in not high incidence country of birth (HIC) population by Local Government Areas (LGA), with relative risk (RR) >1.0 compared to rest of NSW not-HIC population

**LGA**	**Notified TB cases**	**Total not HIC, 2006-2008**	**Annualised TB rate not HIC**	**RR to rest of NSW**	**95% CI**
Metro 1	12	140193	8.6	5.0	2.8 to 8.9
Metro 4	10	234477	4.3	2.5	1.3 to 4.6
Metro 6	14	362850	3.9	2.2	1.3 to 3.8
Metro 9	13	420825	3.1	1.8	1.0* to 3.1
NSW	330	6,231,785	1.8		

* Lower 95%CI 1.02.

There was a strong correlation between the age distribution of TB cases in LGAs with higher rates of TB in the HIC populations, and with the age distribution of TB cases in the same population group in other LGAs (r=0.89, 95%CI 0.73, 0.96).

Direct age standardisation demonstrated significantly higher rates of TB in the age groups 5–19 years, 20–64 years, 65 years and above in the nine LGAs with higher rates of TB in the HIC population. The rate ratio of 2.3 in the 5–19 years age group was significantly higher than other age groups. The 0–4 years age group showed no significant difference to the rest of NSW population with a HIC (Table 4).

**Table 4:** Age standardised TB rates of Local Government Areas with higher rates in high incidence country of birth (HIC) than rest of NSW, 2006-2008

**Age group (years)**	**LGAs with higher TB rates (n=9) in HIC population**			**NSW high incidence c.o.b population excluding the nine high TB rate LGAs**					
	**TB**	**2006-2008 Pop.**	Rate*	**TB**	**2006-2008 Pop.**	Rate*	**Rate ratio**	**95%**	**CI**
								**lower**	**upper**
0-4yrs	1	4,588	21.8	2	10,359	19.3	1.1	0.1	12.5
5-19yrs	34	49,594	68.6	40	133,449	30.0	2.3	1.5	3.6
20-64yrs	399	435,596	91.6	453	940,587	48.2	1.9	1.7	2.2
65+	58	45,815	126.6	83	106,623	77.8	1.6	1.2	2.3
Total	492	533,193	92.3	578	1,191,018	48.5	1.9	1.7	2.1

The nine LGAs with higher rates of TB in the HIC population had a significantly higher proportion of people born in countries with TB rates >100/100,000 population and >300/100,000 population (Table 5).

**Table 5:** Population distribution of the combined Local Government Areas (LGA) with high TB rates (n=9) compared to rest of NSW, by rates of TB >100 and TB>300 in country of birth (c.o.b)

**Area**	**Pop. from c.o.b with TB rate >100**	**Pop. from c.o.b with TB rate <=100**	**p value**	**Pop. from c.o.b with TB rate >300**	**Pop. from c.o.b with TB rate <=300**	**p value**
LGAs with higher rates of TB in HIC pop.	116355 (13.4%)	755065 (86.6%)	p<0.0001	12797 (1.5%)	858623 (98.5%)	p<0.0001
Rest of NSW	249177 (4.4%)	5428566 (95.6%)		44221 (0.8%)	563312 (99.2%)	

The mean length of stay in Australia before diagnosis for people reported with TB in the nine LGAs with higher rates of TB in the HIC population, was 8.9 years with a median of 4 years. This was significantly lower (p<0.0001) than for people with TB from a HIC in the rest of NSW, who had a mean length of stay of 12.1 years and a median of 7 years.

LGAs were ranked by IRSD and correlation explored. There was no correlation between the IRSD and TB rates for HIC populations in LGAs (r=0.11).

## Discussion

New South Wales, Australia, has a low annual incidence of TB, but there are LGAs and population groups with higher rates of TB. In line with other studies [1,8,10] this study demonstrates that TB in NSW predominately occurs in people born in high incidence countries who have migrated to Australia. This study successfully identified LGAs with higher TB rates given the country of birth mix of the local population.

There were nine LGAs where higher TB rates were found in the population born in a HIC. A number of variables were explored to explain this increase. The age distribution of the HIC population was similar in the areas with higher rates compared to the other areas, but the highest differential in rates of TB across areas was found in the 5–19 year age group. This may be a result of a younger population in the sub-groups of people born in a country with very high TB rates. In developed settings TB is often associated with increasing age. The analysis indicated that the age distribution was not a significant factor in explaining the higher rates of TB in the nine LGAs.

The nine LGAs investigated had a higher proportion of the population born in countries where TB rates were >100/100,000 population and >300,000 population. In addition people notified with TB in these nine areas had a shorter length of stay in Australia. These factors are likely contributors to the higher rates of TB found.

No clusters or outbreaks have been reported in the nine LGAs, and health services and particularly TB services are structured in a similar way to surrounding areas.

This study has a number of weaknesses that need to be considered when interpreting the results. Denominators used to calculate the rates are from 2006 Census data; they may not represent the actual number of people with specific country of birth in the remaining years of the study. As the numbers of people born in some countries who reside in NSW are small, a change in their number could have a large effect on country specific TB rates. Thus it was decided to group high incidence and low incidence countries into only two population groups to reduce the effect of possible changes in small numbers from specific countries or regions. While country of birth in the census (denominator) is self-reported, surveillance (numerator) data are more likely to contain health professional reported country of birth and thus sources of information differ.

The estimates of rates are biased by the fact that temporary visitors are included among the cases but are not necessarily enumerated within the base population. Asylum seekers do not appear in the numerator or denominator data. A further weakness is the use of the 2006 Census data to represent the whole study period 2006–2008. In Australia, the Census of the population occurs only every five years with estimates provided in intervening years. Some changes in the mix of the population in LGAs may have occurred during the study period and these changes cannot be measured or accurately estimated.

Using this novel method to analyse local TB rates by country of birth population groups can enable targeting of TB awareness programs and improving access to TB services [4,12,13]. Being alert to the possibility of TB disease is an important step towards its control [19]. Targeted work with medical services, other community based health workers and community groups that serve recent migrants from high TB incidence countries is indicated [13]. Providing accessible education for the population groups and support that addresses access, language and cultural issues is vital within this targeted work [4]. A similar regional analysis conducted in Italy found that TB epidemiology was affected by an increase in the immigrant population and that differentiating between Italy and non-Italy born subjects was worthwhile [20]. The authors argue that this knowledge might help with the planning of tailored and effective prevention and surveillance programmes. A model based on local area analysis may be a useful adjunct to focus work to areas with the greatest need.

Health and immigration policies can impact on TB control. The absence of policies that specifically address poverty-related disadvantages among immigrants makes immigrant populations more vulnerable to the reactivation of their tuberculosis [21]. This is compounded by the racialization and medicalization of TB control policies which can reinforce the unequal distribution of TB burden and lessen the recognition of the importance of poverty [22].

TB control measures, such as contact tracing, can assume new meanings for migrants [23]. Targeting TB strategies carries risks of re-stigmatising population groups especially recently arrived migrants and might create more barriers to TB control [24]. To reduce these risks, understanding of the local epidemiological data about TB needs to be based on crucial factors such as living conditions, life chances and access to affordable and appropriate health care. It is for this reason, and risks of increased stigmatisation, that the specific country of birth has not been reported in this analysis. Focusing TB prevention more on social determinants and less on race will also help to reduce stigma.

This study has focused on TB rates in registered immigrant populations but it should be noted that TB is also an important health issue for undocumented immigrant populations. There is a strong public health rationale for also ensuring early detection and effective treatment of TB until completion in undocumented immigrants. Deporting migrants can cause the discontinuation of their treatment. Immigrants should not be forced to leave the country before adequate diagnosis has been offered, or treatment has been completed [25]. Recommendations to achieve this include health authorities ensuring easy access to low-threshold facilities where undocumented migrants, who are TB suspects, can be diagnosed and treated without giving their names and without fear of being reported to police or migration officials [26].

Four LGAs in NSW had higher TB rates in people born in a high-incidence country and also those not born in a high-incidence country. This may or may not indicate that local TB transmission is occurring. Further detailed epidemiological investigation, including molecular studies, is warranted in these areas.

## Conclusion

In many low TB incidence settings, such as the state of New South Wales Australia, higher rates of TB are reported in the high-incidence country of birth migrant population groups. Analysing local area TB rates and possible explanatory variables, taking into account the different mix of populations by TB incidence in the countries of origin, enables health services to strategically target TB control measures.

Local level epidemiological analysis of TB in people from a high incidence country of birth

## Competing interests

The authors declare that they have no competing interests.

## Authors’ contributions

PM conceived the study, performed the data analysis, participated in the interpretation and drafted the manuscript. DD NS AC contributed to the study design, interpretation of the data and provided critical revisions to the manuscript. All authors read and approved the final manuscript.

## Pre-publication history

The pre-publication history for this paper can be accessed here:

http://www.biomedcentral.com/1471-2458/13/62/prepub
